# Genomic analysis of pancreatic juice DNA assesses malignant risk of intraductal papillary mucinous neoplasm of pancreas

**DOI:** 10.1002/cam4.2340

**Published:** 2019-06-21

**Authors:** Raúl N. Mateos, Hidewaki Nakagawa, Seiko Hirono, Shinichi Takano, Mitsuharu Fukasawa, Akio Yanagisawa, Satoru Yasukawa, Kazuhiro Maejima, Aya Oku‐Sasaki, Kaoru Nakano, Munmee Dutta, Hiroko Tanaka, Satoru Miyano, Nobuyuki Enomoto, Hiroki Yamaue, Kenta Nakai, Masashi Fujita

**Affiliations:** ^1^ Department of Computational Biology and Medical Science The University of Tokyo Chiba Japan; ^2^ Human Genome Center, The Institute of Medical Science The University of Tokyo Tokyo Japan; ^3^ Laboratory for Cancer Genomics RIKEN Center for Integrative Medical Sciences Yokohama Japan; ^4^ Second Department of Surgery Wakayama Medical University Wakayama Japan; ^5^ First Department of Internal Medicine University of Yamanashi Yamanashi Japan; ^6^ Department of Surgical Pathology Kyoto Prefectural University of Medicine Kyoto Japan; ^7^ Laboratory of DNA Information Analysis, Human Genome Center, Institute of Medical Science The University of Tokyo Tokyo Japan

**Keywords:** biomarkers, cell‐free nucleic acids, exome, pancreatic juice, pancreatic neoplasms

## Abstract

Intraductal papillary mucinous neoplasm (IPMN) of pancreas has a high risk to develop into invasive cancer or co‐occur with malignant lesion. Therefore, it is important to assess its malignant risk by less‐invasive approach. Pancreatic juice cell‐free DNA (PJD) would be an ideal material in this purpose, but genetic biomarkers for predicting malignant risk from PJD are not yet established. We here performed deep exome sequencing analysis of PJD from 39 IPMN patients with or without malignant lesion. Somatic alterations and copy number alterations (CNAs) detected in PJD were compared with the histologic grade of IPMN to evaluate their potential as a malignancy marker. Somatic mutations of *KRAS*, *GNAS*, *TP53*, and *RNF43* were commonly detected in PJD of IPMNs, but no association with the histologic grades of IPMN was found. Instead, mutation burden was positively correlated with the histologic grade (*r* = 0.427, *P* = 0.015). We also observed frequent copy number deletions in *17p13* (*TP53*) and amplifications in *7q21* and *8q24* (*MYC*) in PJDs. The amplifications in *7q21* and *8q24* were positively correlated with the histologic grade and most prevalent in the cases of invasive carcinoma (*P* = 0.002 and 7/11; *P* = 0.011 and 6/11, respectively). We concluded that mutation burden and CNAs detected in PJD may have potential to assess the malignant progression risk of IPMNs.

## 1 INTRODUCTION

Intraductal papillary mucinous neoplasms (IPMNs), described for the first time in 1982 by Ohashi et al.^1^, ^2^ and posteriorly defined by the World Health Organization, are pancreatic tumors with unique characteristics including hyper‐production of mucin in tall columnar epitheliums, and dilatation and papillary growth inside the pancreatic ducts.^3^, ^4^, ^5^, ^6^ After the establishment of its diagnosis, the incidence of IPMN has been rapidly increasing, and it is now understood that IPMN can be classified along the spectrum of adenoma to carcinoma, and it can be a precursor of pancreatic cancer.^7^ Its prognosis, compared with pancreatic ductal adenocarcinoma (PDAC), is relatively better after surgical resection.^8^ However, IPMN is histologically very heterogeneous and some parts can progress from low to high‐grade dysplasia and finally to invasive adenocarcinoma, which shows as poor prognosis as PDAC.^9^ Furthermore, PDAC is sometimes coincident with IPMN.^10^ Therefore, it is clinically important to assess the risk of pancreatic cancer progression and the development in IPMNs in order to take the decision of tumor resection.^11^, ^12^ There are several guidelines and recommendations that support the assessment of the risk for malignancy of IPMN by imaging methods such as magnetic resonance imaging/cholangiopancreatography, but there is still some room for improvement. Now it is essential to develop other less‐invasive approaches than biopsy or surgery to assess the malignant potential of IPMNs based on biomarkers or genomic alterations.

Cell‐free DNA shed by tumor cells is a rich source of tumor‐specific biomarkers and genomic analysis, as shown in studies on cell‐free DNA derived from plasma,^13^, ^14^, ^15^ urine,^16^ and cerebrospinal fluid (CSF).^17^ Tumor‐derived DNA can be effectively captured and analyzed in anatomically relevant fluids, such as urine in bladder cancer, and endocervical fluid for gynecological tumors.^18^ Pancreatic juice could be an ideal material for assessing malignancy risk of IPMN and pancreatic tumors as well. In fact, cytological test in pancreatic juice can inform of the malignant potential of IPMN.^19^ Some hospitals screen patients with IPMN by endoscopic retrograde cholangiopancreatography (ERCP) and cytological test. Moreover, due to its liquid nature, pancreatic juice has the potential to overcome the heterogeneity problem inherent to biopsy specimens from IPMN. Molecular analysis and characterization of pancreatic juice may provide direct evidence of malignant IPMN, thereby complementing the imaging.

Regarding genomic alterations and biomarkers, several genes and pathways are commonly altered in IPMNs. *KRAS* oncogene mutation has been described in different frequencies (31%‐86%) although its incidence does not correlate with the levels of histologic grade or dysplasia.^20^ *GNAS*, another oncogene, has been reported as mutated in codon 201 in more than half (61%) of their patients with IPMN 21 and has been previously detected by droplet digital polymerase chain reaction in cfDNA obtained from blood samples.^22^ Oncogenes such as *BRAF*, *PI3KCA,* and *TERT*, tumor suppressor genes such as *CDKN2A* or *TP53*, as well as pathways such as the Sonic Hedgehog signaling pathway have also been reported as altered in some studies.^12^, ^23^, ^24^, ^25^

Until now, several studies had analyzed pancreatic juice for driver mutations such as *KRAS* and *GNAS*.^25^, ^26^ However, it is still unclear whether these mutations are useful as markers of IPMN malignancy. More comprehensive genomic analysis of pancreatic juice cfDNA (PJD) may help to discover better markers of malignancy and to overcome the intratumoral heterogeneity of IPMN. Here, we performed deep exome sequencing of PJDs from 40 IPMN patients and found that mutational burden and copy number alterations (CNAs) detected in PJDs could evaluate the malignancy of IPMN.

## 2 MATERIALS AND METHODS

### 2.1 Clinical samples

Pancreatic juice and blood samples from 40 IPMN patients were obtained in Wakayama Medical University Hospital and Yamanashi University Hospital. All subjects agreed with informed consent to participate in the study and the Institutional Review Boards at RIKEN and the two hospitals approved this work. The pancreatic juice samples were collected by ERCP or endoscopic nasopancreatic drainage (ENPD). All of these patients underwent surgical resection for these tumors after obtaining its pancreatic juice, and the pathological description of these resected specimens was reviewed by experienced pathologists in Kyoto Prefectural University of Medicine. Their clinicopathological features are shown in Table 1 and Table S1. Regarding their pathology, eight (20%) had low‐grade dysplasia (LGD), 20 (50%) had high‐grade dysplasia (HGD), whereas 12 (30%) had invasive carcinoma (INC).

**Table 1:** Summary of clinical information of IPMNs

Sex	Male	23
	Female	16
Histological grade	Low‐grade dysplasia	8
	High‐grade dysplasia	20
	Invasive carcinoma	11
Subtype	Intestinal	11
	Gastric	21
	Pancreatobiliary	4
	NA	3
Macroscopic type	Branch duct	13
	Main duct	11
	MIX	15
Tumor location	Head	21
	Head‐tail	2
	Tail	1
	Body‐tail	12
	Body	3

### 2.2 Exome sequencing of PJD

**Figure 1:**
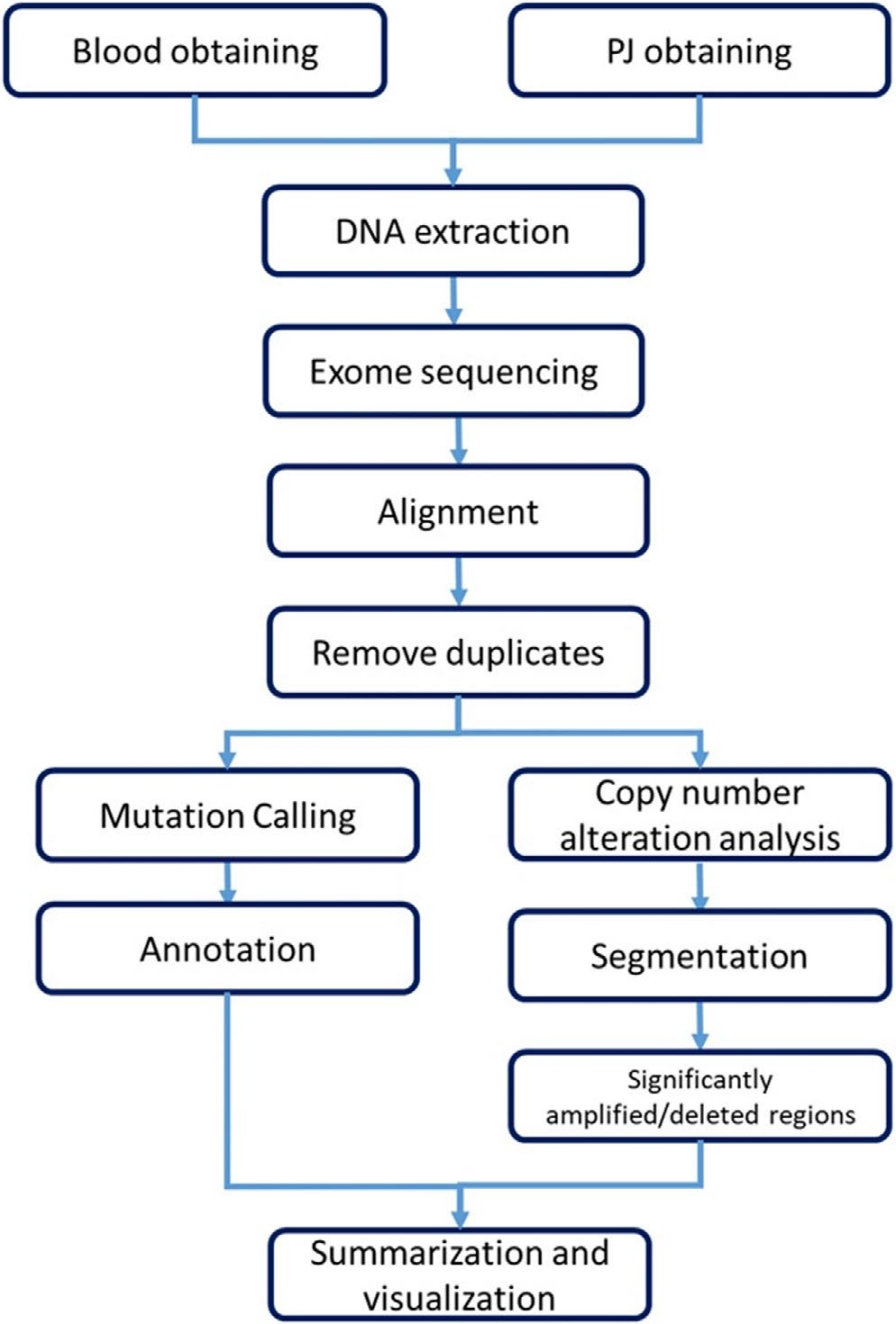
Workflow of deep exome sequencing analysis for PJD. The pipeline divides into two analysis: the mutation calling and the CNA analysis. Then, the outputs were gathered for visualization

Analysis flowchart of this study is shown in Figure 1. Cells or cell‐debris in pancreatic juice was removed by the centrifugal separation and the cell‐free DNA was extracted from 0.5 to 1 mL pancreatic juice samples by using QIAamp Circulating Nucleic Acid Kit (Qiagen), following its protocol for cfDNA extraction from plasma after scaling up the volume by adding saline. Normal DNA was extracted from lymphocyte in blood by QIAamp DNA Blood Kit (Qiagen). The libraries were prepared with KAPA HyperPrep Kit (Kapa Biosystems) using 5‐10 ng PJDs without any fragmentation process and 50ng lymphocyte normal DNAs after fragmentation process. The exome capture was performed with SureSelect Human All Exon V5 (Agilent Technologies), and sequencing was performed with Illumina HiSeq2500 SBS V4. Sequencing coverage was 200‐300× for PJD and 100× for normal lymphocyte DNA. Reads were mapped to GRChr37 using BWA and duplicates from PCR were removed using Picard.^27^

### 2.3 Mutation calling

For the analysis of single nucleotide variation and INDELs, we used the tool Genomon2 Fisher mutation call.^28^ The minimum depth for the mutation call was 8, the minimum map quality was 20, and the minimum base quality was 15. Variants with less than 3 reads were filtered. The disease minimum allele frequency chosen for our study was 0.05, the control maximum allele frequency was 0.1 and the Fisher mutation call threshold was set to 0.05. The detected variants were annotated using Annovar^29^ and then summarized and visualized using the R package Maftools.^30^

The generation of 8‐oxoguanine (OxoG) is one of the most common artifacts produced during the library construction. The OxoG alteration can be caused by the effect of heat, storage, shearing, contamination of metals, or its combined effect, leading to C > A/G > T transversion commonly formed in the middle base of CCN triplets.^31^ Three lines of evidence showed that three PJD samples (W18, W21, and W24) were highly affected by the OxoG artifact. Firstly, the variant allele frequency (VAF) of the three samples was lower than the other samples (Figure S1). It is known that mutations caused by the OxoG artifact have a lower VAF.^31^ Secondly, the oxidation error rates computed by Picard CollectOxoGMetrics^27^ were strikingly high in the three samples (Figure S2). Finally, the trinucleotide mutation patterns of the three samples had the characteristic configuration of OxoG artifacts (Figure S3). Because of all these evidences, we excluded these three samples from the SNV analysis.

### 2.4 CNA analysis from exome sequencing data of PJD

The copy number variation analysis was carried out using Varscan2.^32^ The ratio of tumor reads and normal reads, required for normalization, was obtained by the SAMtools function flagstats. For the CopyNumber function, the p‐value threshold was 0.005, minimum segment size was 100 bases, and the maximum segment size was 100 000. The output obtained was then used as the input for the CopyCaller function of Varscan2. Circular binary segmentation (CBS) was posteriorly performed on this output by using the R package DNAcopy^33^ by which we undid splits that were not at least 3 standard deviations apart. The segmentation from CBS obtained was analyzed using GISTIC2.0,^34^ in order to find significant amplified or deleted regions among the samples. The threshold for copy number amplifications was 0.2. Copy number segments with a log2 ratio above the value was considered amplified. Likewise, the threshold for copy number deletions was set to −0.2. The confidence level to calculate the region containing a driver was set to 0.90.

One issue that should be taken into account in CNA analysis is the potential appearance of samples with high variance in their segmentation, leading to blurry and unstable outcomes. In these samples, the lack of a stable neutral value makes them very difficult to normalize/adjust, set thresholds for amplifications and deletions, and eventually, this high variance affects the performance of CNA analysis tools like GISTIC2.0. In order to avoid this kind of outcome, we proceeded to evaluate the level of instability by obtaining the residual variance of each sample. (Table S2). After analyzing the results of this evaluation, four samples (W20, W22, W23, and Y20) were removed from our dataset due to their high residual variance.

The exome capture was done with SureSelect Human All Exon V5, which bounds our CNA to exome regions. For this reason, all the outputs from Varscan2 located exclusively in off‐target regions of the exome capture should be considered untrustworthy. To improve the results of our analysis, we removed these intron‐only–generated regions impossible to have been captured by our kit. After this filtering, we re‐fused consecutive segments that shared the same log2 ratio. By doing so, we were able to generate a much cleaner and reliable input for GISTIC2.0.

## 3 RESULTS

### 3.1 Deep exome sequencing of PJD

To identify somatic mutations of IPMNs in PJD, we collected pancreatic juice and blood of 40 IPMN cases and extracted DNA from these samples. We constructed next generation sequencing libraries from a small amount of PJD (5‐10 ng) and performed deep exome sequencing of PJD together with blood DNA for 40 IPMN cases. We excluded one case because it was diagnosed as PDAC and not IPMN after resection, and we analyzed exome sequence data of PJDs of 39 IPMNs. The median number of sequence reads were 163 million reads for PJD, and the median depth on target was 168× after duplication removal. For blood samples, the median number of sequence reads were 82 million reads, and the median depth on target was 112× after duplication removal (Table S3). The median of duplication rate was 18.7% in exome of PJD and was higher than that in blood DNA exome (5.4%). This may be caused by low‐input DNA for the library preparation (Figure S4).

**Figure 2:**
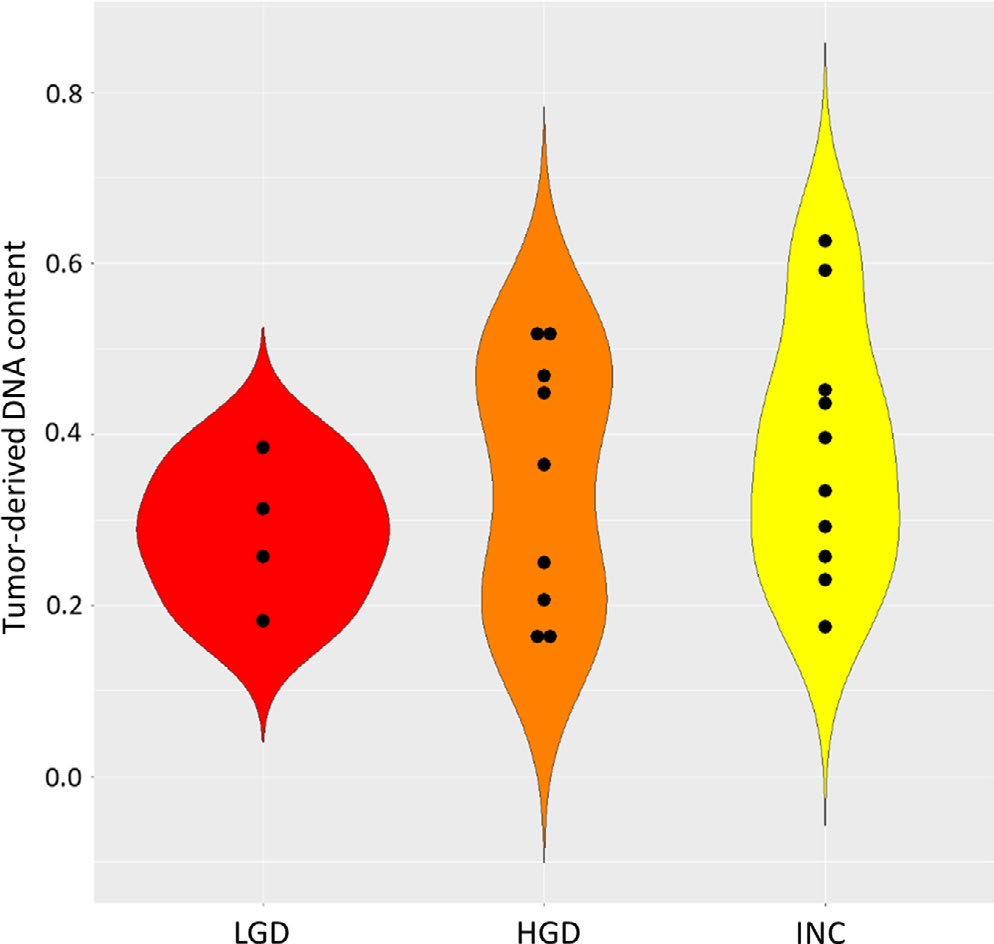
Violin‐plot of the median of predicted tumor‐derived DNA content in PJD by the histologic grade. PJD samples with more than 10 mutations were shown along with their IPMN grade. No correlation of tumor‐derived DNA content with the grade was found (*P* = 0.148)

We estimated the content of tumor‐derived DNA in PJD by doubling the median VAF in PJD samples. To reduce statistical error, we limited the analysis to 23 PJD samples that had more than 10 somatic SNV/INDEL alterations. The median of predicted tumor‐derived DNA content among those 23 PJD samples was 33.4% (Figure 2; Table S4), indicating higher content of tumor‐derived DNA in pancreatic juice of IPMN patients than those obtained in CSF from patients with central nervous system metastases.^35^ No significant correlation of tumor‐derived DNA content with the histologic grade of IPMN was found (*P* = 0.265) (Figure 2).

### 3.2 Mutation burden in PJD was associated with histologic grade of IPMN

**Figure 3:**
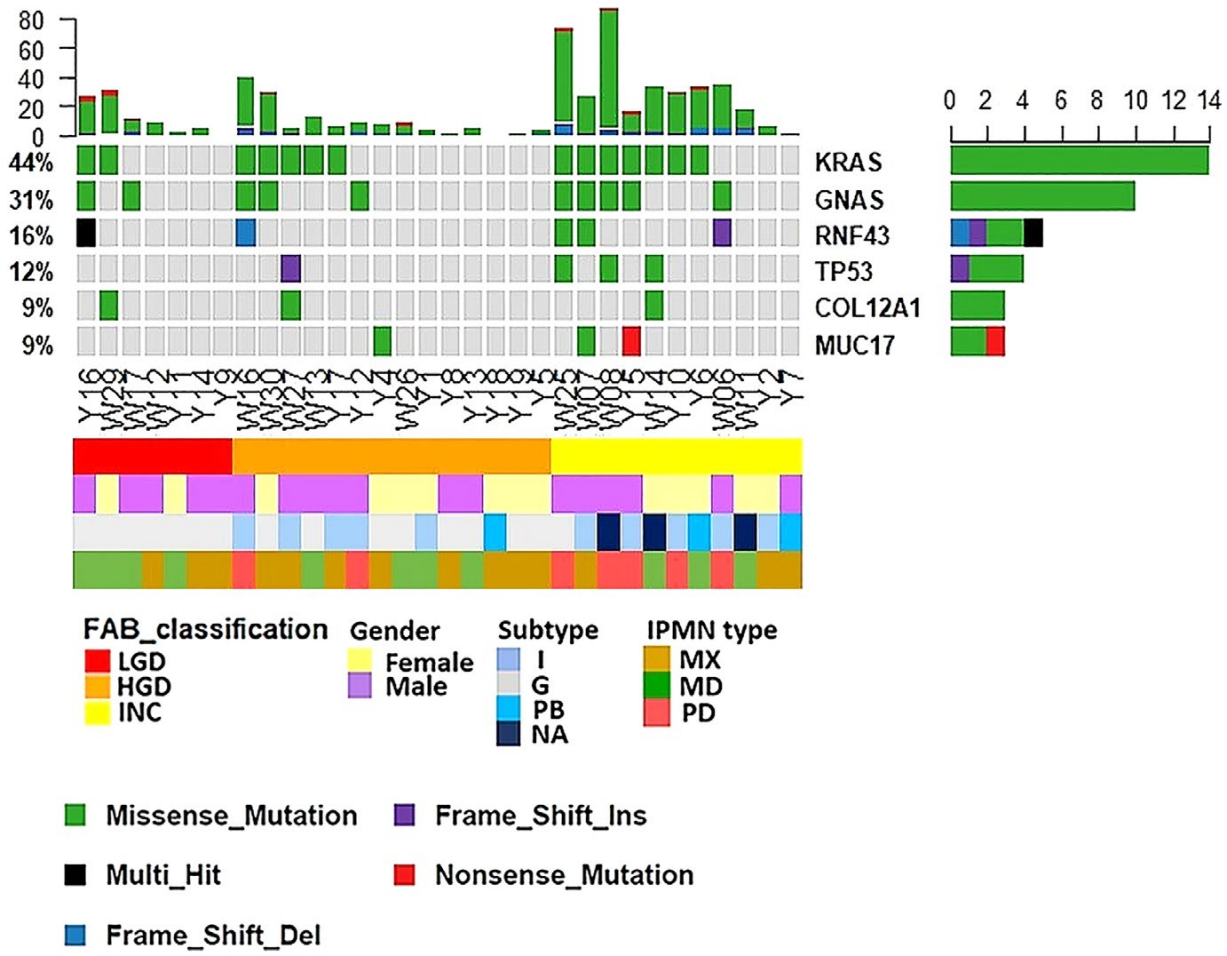
Somatic mutations detected in PJD and the histologic grade of IPMN. The figure shows somatic mutations present in our dataset. Below the table, clinical features of the samples are included. Genes with at least two mutations were included. The most common mutations were in *KRAS* and *GNAS*, but no significant association with the histologic grade was found. Four of five *TP53* mutations were found in INC samples

After excluding the case that was identified as PDAC, seven cases with OxoG and/or high residual variance were removed from the 39 cases, and therefore we called somatic mutations for the remaining 32 cases. Totally, 579 somatic nonsynonymous mutations affecting 518 genes were detected (Figure 3). Among them, 494 mutations (85.32%) were missense mutations, 30 (5.18%) were frame‐shift deletion, 25 (4.32%) were nonsense mutations, 15 (2.59%) were frame‐shift insertions, 10 (1.73%) were splice‐site mutations and five (0.86%) were in‐frame deletions. The median number of total mutations per sample was 24 (0.471/Mb), which indicates that the mutation burden of IPMN calculated from PJD has lower mutation rate compared to pancreatic cancer.^36^

Interestingly, the whole‐exome mutation burden in PJD was associated with the histologic grades of IPMN (Figure 3). The median number of mutations was 16 in LGD, 19.5 in HGD, and 64 in INC Spearman correlation coefficient between the histologic grade of IPMN and the number of mutations was 0.427 (*P* = 0.015). This result indicated that the mutation burden in PJD might serve as a marker for malignant potential of IPMN. In contrast with this association, the mutational signature had a similar distribution irrespective of grades (Figure S5). The association of mutations with other clinical features of IPMNs is shown in Figure S6A. *GNAS* mutation was associated with male (*P* = 0.019) and with main duct type (*P* = 0.018), consistently with previous reports.^2^, ^37^

*KRAS* and *GNAS* were most frequently mutated (14 and 10 samples; 43.75% and 31.25%, respectively), which was also consistent with previous studies.^23^, ^25^, ^37^, ^38^ *RNF43* was mutated in five samples (15.63%) whereas TP53 was altered in four (12.5%). *RNF43* is a RING‐type E3 ubiquitin ligase whose loss‐of‐function mutation can activate the Wnt/β‐catenin signaling and is commonly mutated in IPMN and in other tumors.^25^, ^39^ Other recurrently mutated genes were *COL12A1*, which encodes the alpha chain of type XII collagen, and *MUC17* which encodes a protective membrane‐bound mucin to gut epithelial cells and has been reported as highly expressed in PDAC as well as a marker for poor prognosis.^40^ Both *COL12A1* and *MUC17* were mutated in three samples (9.38%).

To verify the presence of mutations detected in pancreatic juice, we dissected several small lesions of IPMN from two FFPE specimens (Cases W14 and W25), which were assigned by the experienced pathologist in HE‐stained slides. DNA was extracted from these small lesions and the mutations of *TP53* were amplified by PCR and analyzed by capillary sequencing. As shown in Figure S7, the two *TP53* mutations p.H214R in W14 (A) and p.R248W in W25 (B) were validated in their malignant lesions (INC), but not in the benign lesions (LGD). We amplified regions of 192 somatic mutations (including SNVs and INDELs) and the *KRAS* and *GNAS* hotspot mutations from DNAs extracted from the corresponding FFPE tumor samples and validated them by deep sequencing and capillary sequencer. Altogether, 120 of 181 mutations were validated (65.57%), being the median of the VAF 0.071, giving a representation of the tumor purity and heterogeneity of the pool.

### 3.3 CNAs in PJD were associated with the histologic grade of IPMN

**Figure 4:**
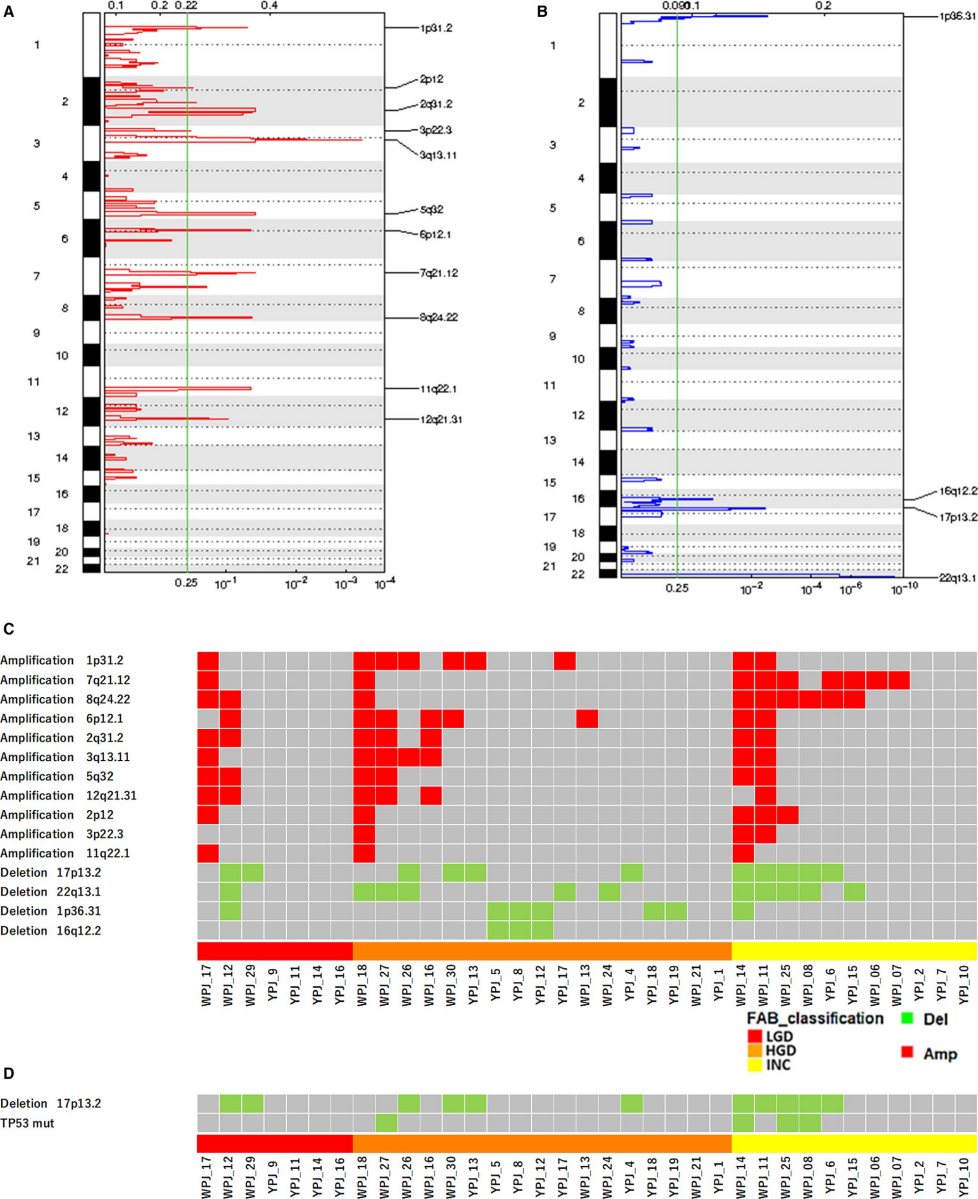
Summary of copy number alterations detected in PJD from IPMNs. (A) Eleven significantly amplified regions detected by GISTIC2.0. (B) Four significantly deleted regions detected by GISTIC2.0, including *17p13.2* (*TP53*). (C) Significantly amplified regions (red) and significantly deleted regions (green) by patient and by grade are shown. The *7q21* amplification and *8q24* amplification (*MYC*) in PJDs showed significant association with INC (*P* = 0.002 and *P* = 0.011, respectively, by Fisher's exact test). (D) Co‐occurrence of *TP53* mutation and *17p13.2* deletion: The co‐occurrence of both alterations are found on PJD samples with INC

We called somatic CNAs from PJD exome data and detected 11 significantly amplified regions (*1p31.2, 2p12, 2q31.2, 3p22.3, 3q13.11, 5q32, 6p12.1, 7q21.12, 8q24.22, 11q22.1,* and 1*2q21.31*) and four significantly deleted regions (*1p36.31, 16q12.2, 17p13.2* and *22q13.1*) (Figure 4A, 4). When analyzing the gene ontology of the genes in regions significantly deleted by amiGO,^41^ we observed an enrichment in DNA related processes such as DNA modification (GO:0006304), DNA deamination (GO:0045006) (Table S5). No enriched processes were found in amplified regions.

The detected CNAs were evaluated for their potentials as malignancy markers (Figure 4C). We observed a significant association between the histologic grade and amplification of two regions (*7q21.12* and *8q24.22*). The *7q21* amplification was found in one of seven (14.26%) in LGD, one of 17 (5.88%) in HGD, seven of 11 (63.64%) in INC (*P* = 0.002 by Fisher's exact test). *KIAA1324L* is located in this amplified region, but it has not been reported as associated with IPMNs and pancreatic cancer. The *8q24* amplification was found in two of seven (28.57%) in LGD, one of 17 (5.88%) in HGD, six of 11 (54.55%) in INC (*P* = 0.011). *MYC* is located in the *8q24* amplified regions in IPMNs (Table S6), which is one of the most frequently altered genes by CNAs in pan‐cancer analysis 42, 43 and whose amplification has been found in pancreatic acinar cell carcinomas.^44^, ^45^ The deletion region *17p13,* which contains *TP53* (Table S7) was also found to co‐occur with *TP53* point mutations in all of the malignant samples (INC) with *TP53* mutations (Figure 4D), which might be consistent with the two‐hit theory of tumor suppressor genes. The association of CNAs in PJD with other clinical features of IPMNs is shown in Figure S6B.

We posteriorly applied GISTIC2.0 to benign (LGD) and malignant samples (HGD and INC) separately, leading us to a more grade‐associated outcome. In benign samples, we observed two significantly deleted regions (*11p15.5* and *11q21*), whereas in malignant samples, a significantly amplified band was found (*3q13.11*) and three regions were significantly deleted (*1p36.33, 17p13.2* and *22q13.1*). The existence of *17p13.2* among these deleted regions reinforces the association of this lesion to the development of malignancy (Figure S8). As for the fractions, nine of 28 malignant samples (32.14%) contained a deletion of this region; four of nine (44.44%) were HGD and five of nine (55.55%) were INC samples.

## 4 DISCUSSION

We here succeeded in genomic profiling of heterogeneous IPMNs by comprehensive genomic analysis of PJD. Pancreatic juice is a useful source for genetic profiling of IPMN and pancreatic tumors; it is less invasive than biopsy and deals better with the heterogeneity of the tumor. With this method, we not only detected critical mutations such as *KRAS* and *GNAS*, known markers in IPMN that prove to a certain extent the reliability of this sample source, but also found out that the mutation burden is significantly correlated with IPMN histologic grade which has been also previously reported in late stages of gynecologic cancers.^46^, ^47^, ^48^ This correlation of mutation burden with histologic grade can be associated not only with the development of malignancy in IPMN, but also with the genomic heterogeneity inherent to advanced tumor samples and our distinct sampling method; different cell populations might possess singular mutations that could go undetected by traditional sampling, but they may be able to be observed on its entirety by the use of liquid biopsies, which have the potential to gather information from all the cell populations.

We as well found multiple regions significantly deleted and amplified in our dataset. Finding *TP53* deleted in the malignant state of the IPMN also reinforced the reliability of our method. More interestingly, two of the regions (*7q21.12* and *8q24.22*) were significantly amplified in malignant samples, which lead us to discover the association of *MYC*, a known marker of malignancy, with the development of IPMN. The amplification and deregulation of *MYC* have been reported in numerous cancer studies, and its key role in metabolism, cell cycle, the biogenesis of ribosomes, and consequently, in cell growth and proliferation makes it the perfect target and marker for malignancy in IPMN detection by PJD.^49^, ^50^, ^51^, ^52^ This finding, combined with the correlation of mutation burden with grade, has strong potential to predict the progression of IPMNs to malignancy, and exome sequencing or whole genome sequencing analysis of PJD is useful to assess the malignant risk of IPMNs. However, due to the poor quality of the paraffin block DNAs, as well as the genetic and histological heterogeneity of IPMN, the validation of CNA in the IPMN tissues was inconclusive in our study, and it will be required to validate this association of CNAs in further fresh sample cohort or by other methods.

Although we demonstrated the proof‐of‐concept of PJD exome testing, its clinical deployment has to overcome several issues. Firstly, the clinical procedure to obtain pancreatic juice remained controversial due to being prone to producing pancreatitis. It has not been recommended in international consensus guidelines of 2012 for the management of IPMN.^53^ Further study will be required to assess the risk and benefit of the PJD exome testing. Secondly, we had to exclude some of our PJD samples because their exome data contained high levels of noise. Three samples were affected by artifacts in SNVs that resemble OxoG, whereas four samples had extreme fluctuations in their copy number segments. These blurry segmentations might not actually be caused by an artifact, but by the heterogeneity of tumor subpopulations in certain samples and the capacity of liquid biopsies to overcome this heterogeneity. Different subpopulations with different alterations in different proportions could cause a mosaic‐like outcome, complicating the evaluation of CNA. Despite its potential interest, we decided our results would be more robust with the removal of this instability. However, the yield should be improved through protocol optimization. Potential strategies are addition of antioxidants to prevent OxoG, and whole genome sequencing to reduce copy number noise.

## 5 CONCLUSION

We detected somatic SNV/INDELs and CNAs through exome sequencing of 39 PJDs. Whole‐exome mutation burden and CNAs in *7q21* and *8q24* in PJD were associated with the histologic grade of IPMNs as well as *TP53* alterations. Testing these makers in pancreatic juice would provide a less‐invasive measure to predict malignancy risk of IPMN.

## CONFLICT OF INTEREST

The authors have no conflict of interest to declare.
