# Supplementary material for: Genomic analysis of pancreatic juice DNA assesses malignant risk of intraductal papillary mucinous neoplasm of pancreas
Source: Cancer Med. 2019 Jun 21;8(10):4565–73. doi: 10.1002/cam4.2340 (PMC6712468; doi:10.1002/cam4.2340)

**Figure S1:** Variant Allele Frequency distribution of Wakayama and Yamanashi samples. Samples with less than 10 mutations were filtered out. The number of mutations differs a lot among the samples, and the variant allele frequency is generally low, but this becomes even more noticeable in the samples affected by the OxoG artifact (W18, W21 and W24). These samples contain a high number of mutations as well as the characteristic C>A mutation pattern.


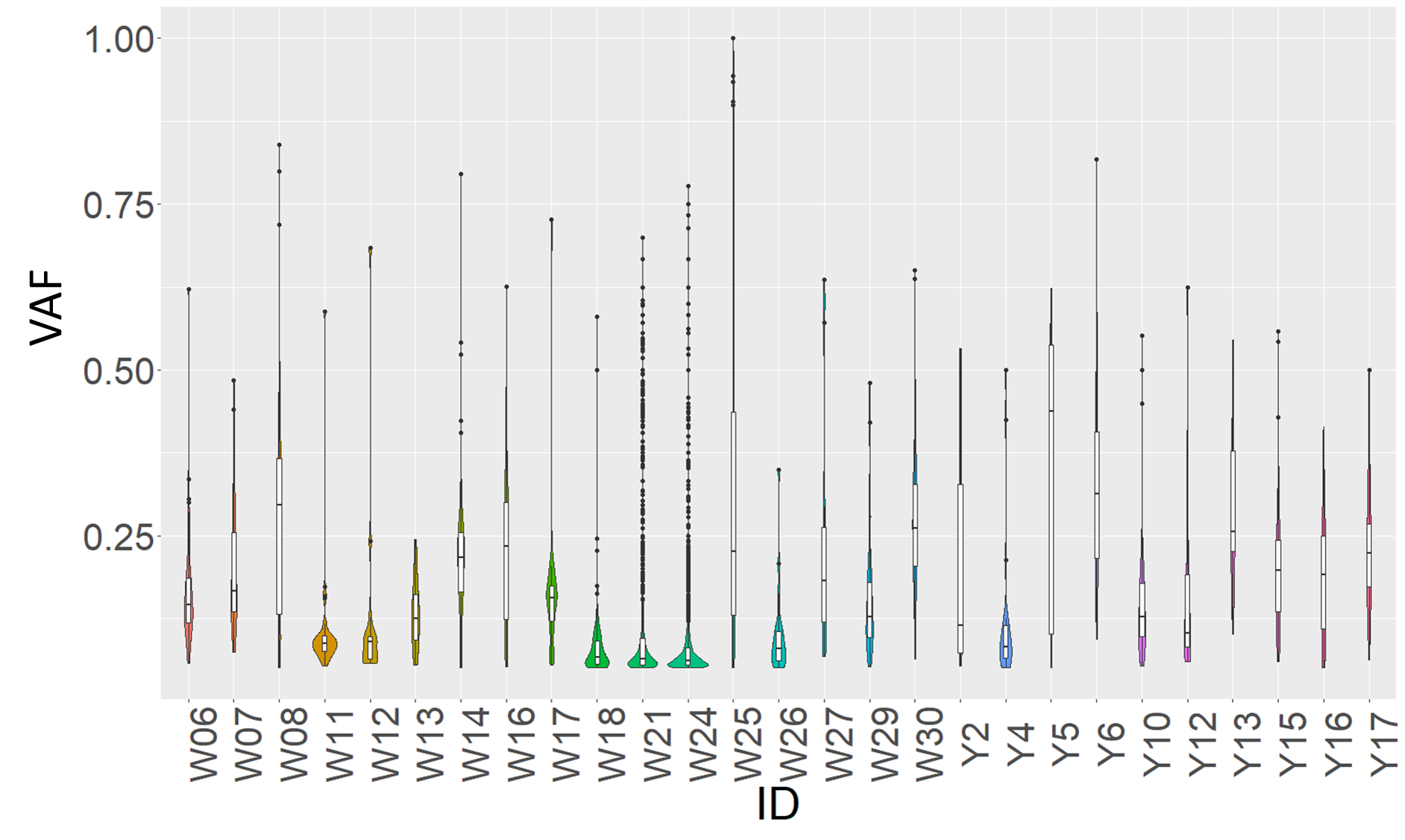


**Figure S2: Results from OxoGmetrics by Picard.** A Shows the oxidation error rate whereas B shows the oxidation Q value per sample. Samples affected by the OxoG artifact show a strikingly different error rate and Q value distribution compared to the others.

**
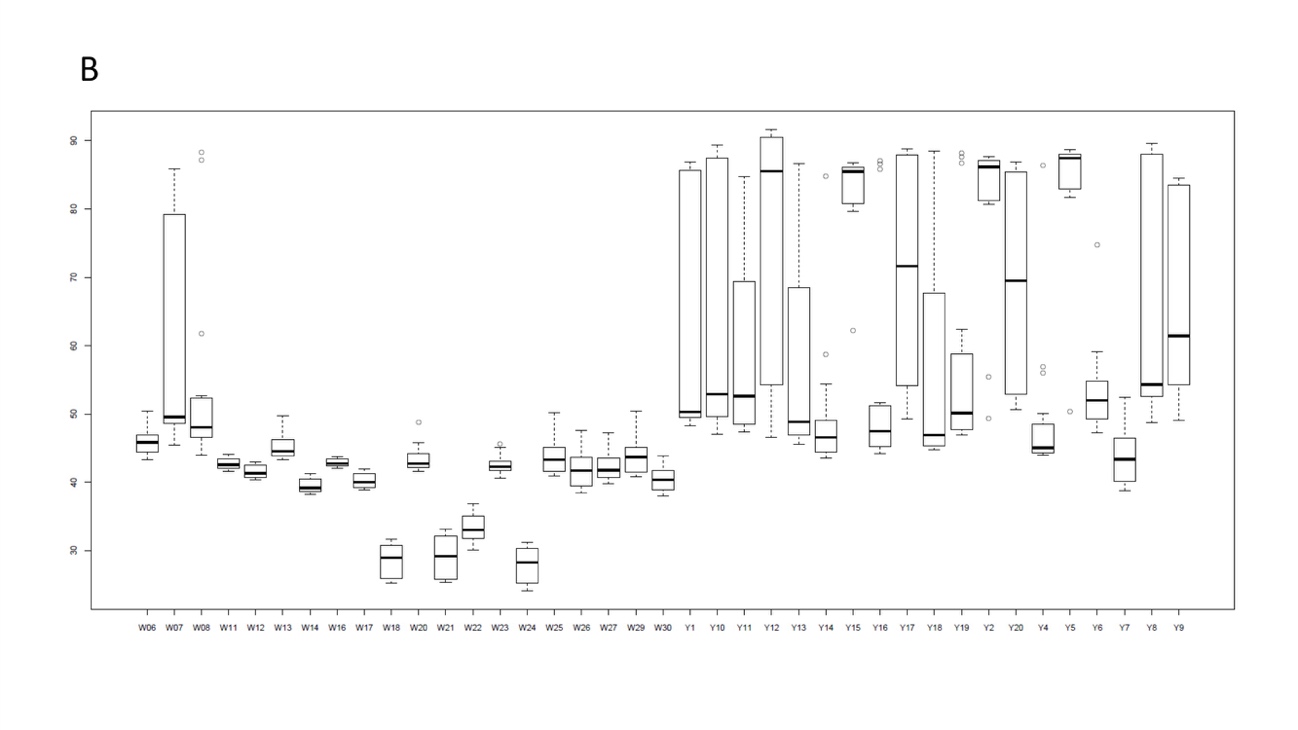

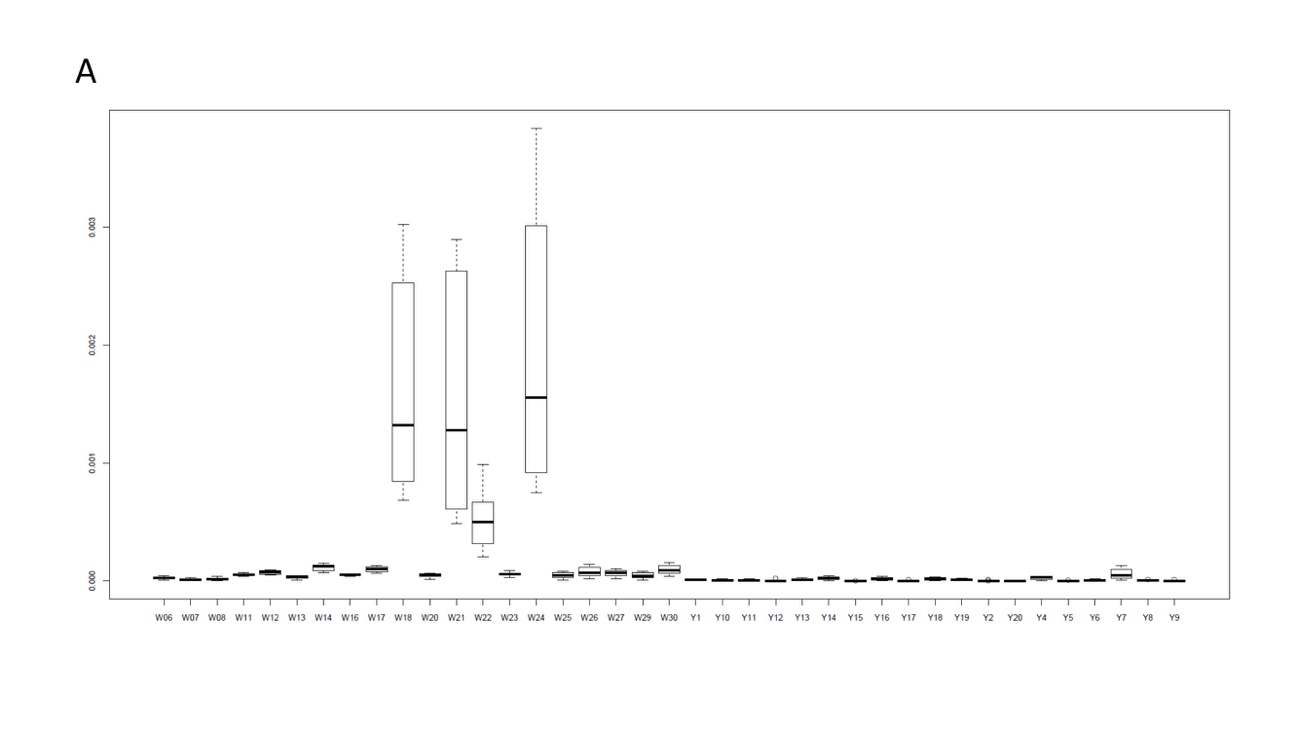
**

**Figure S3: Mutation pattern in samples W18, W21 and W24, affected by OxoG artifact.** Samples affected by the OxoG artifact have a high number of mutations, as well as an augmented number of C>A mutations that corroborates the assumption of them being altered.


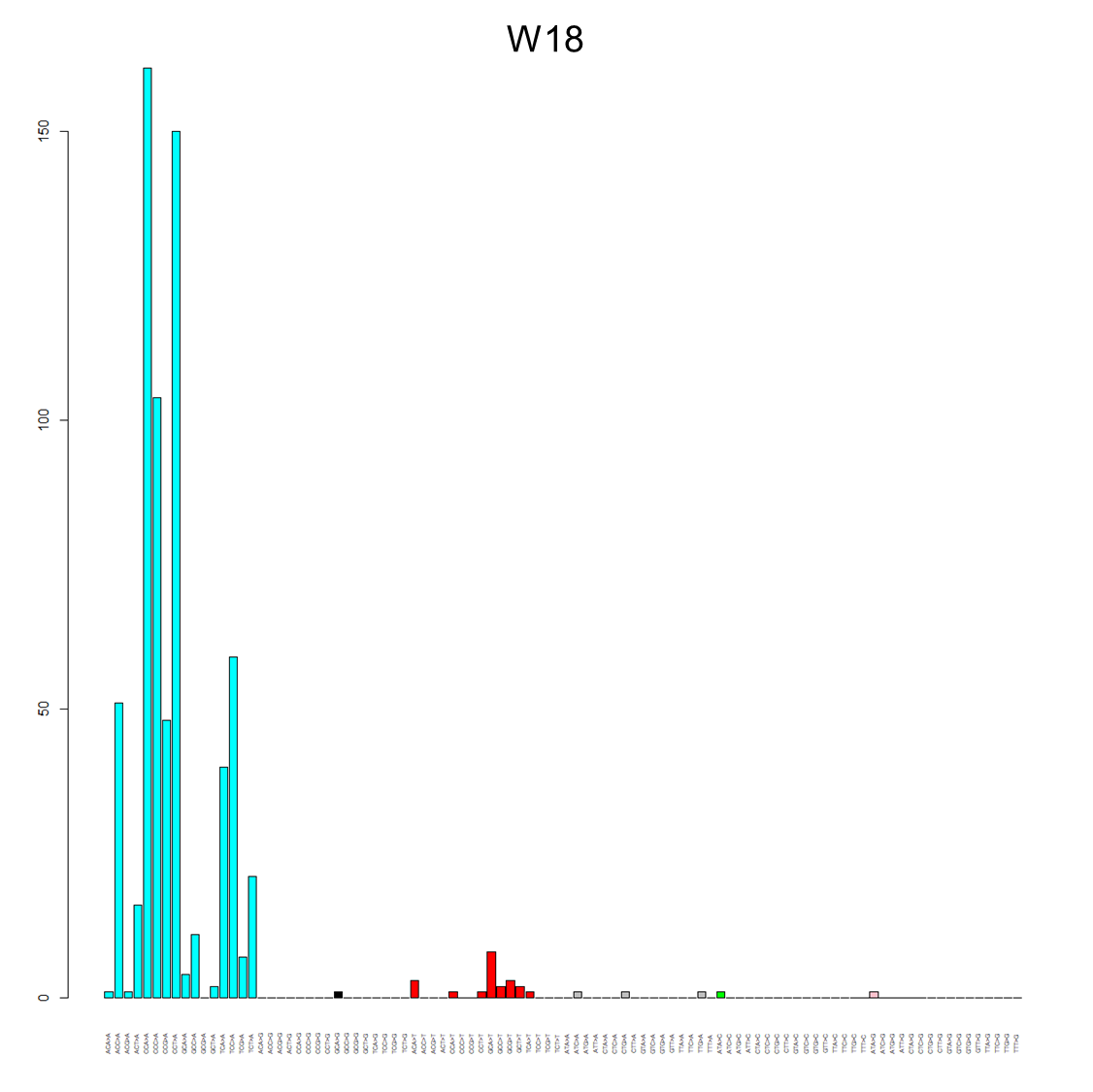

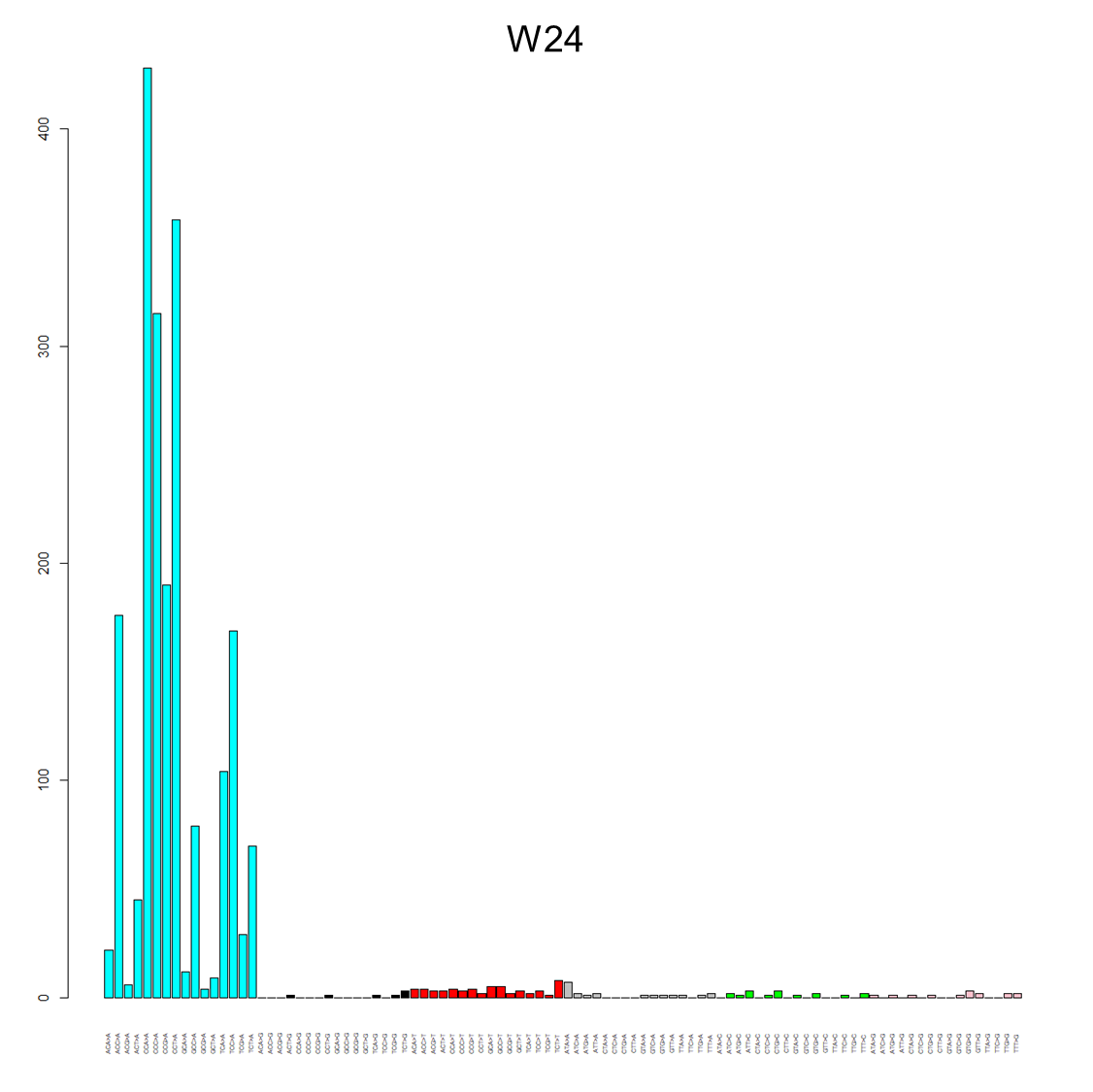

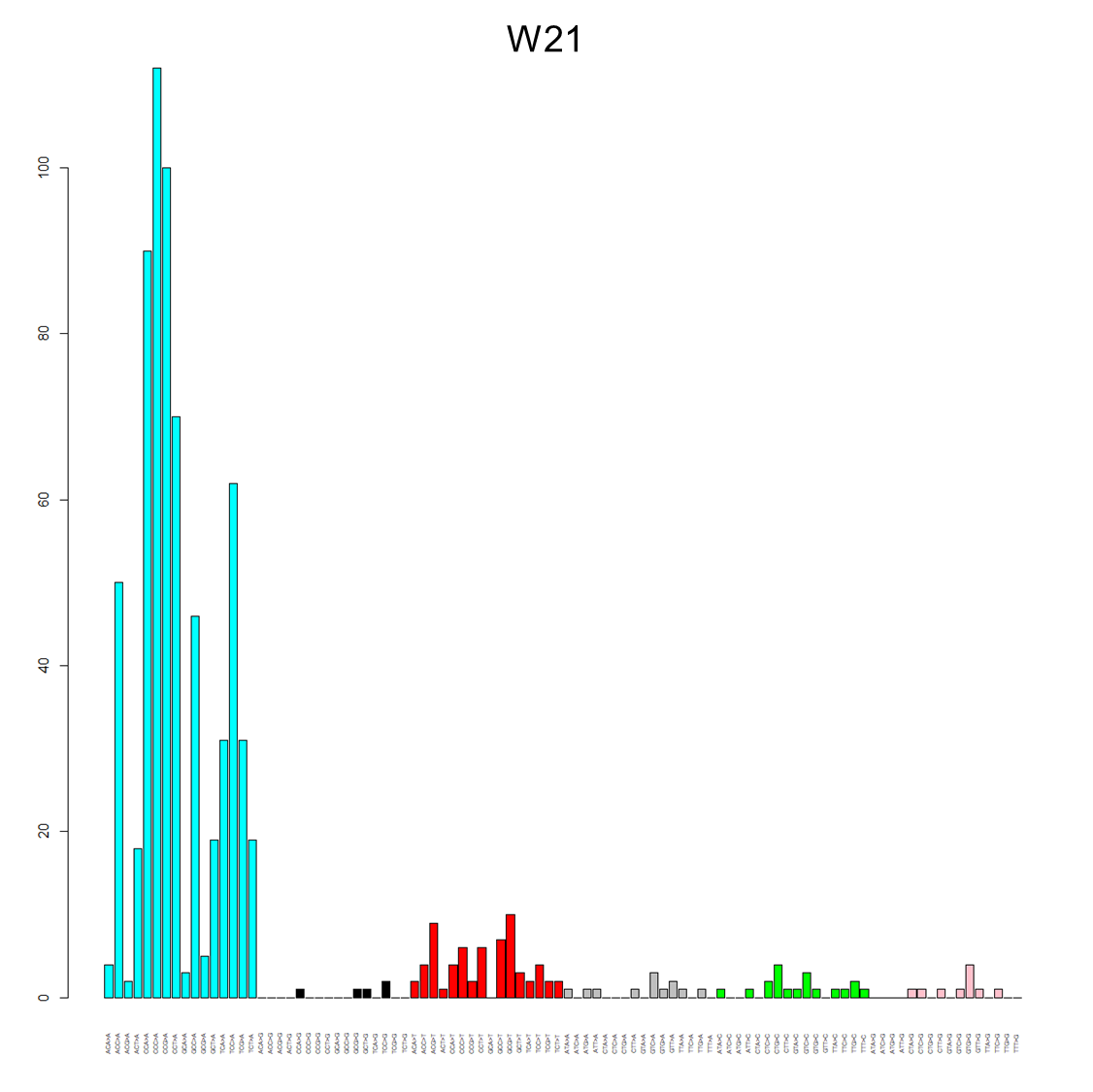


**Figure S4: Duplicate reads:** Duplicate reads content per sample in (A) Blood samples and (B) Pancreatic Juice samples. The median of duplication reads in PJD was higher than the median in the blood DNA samples.

**
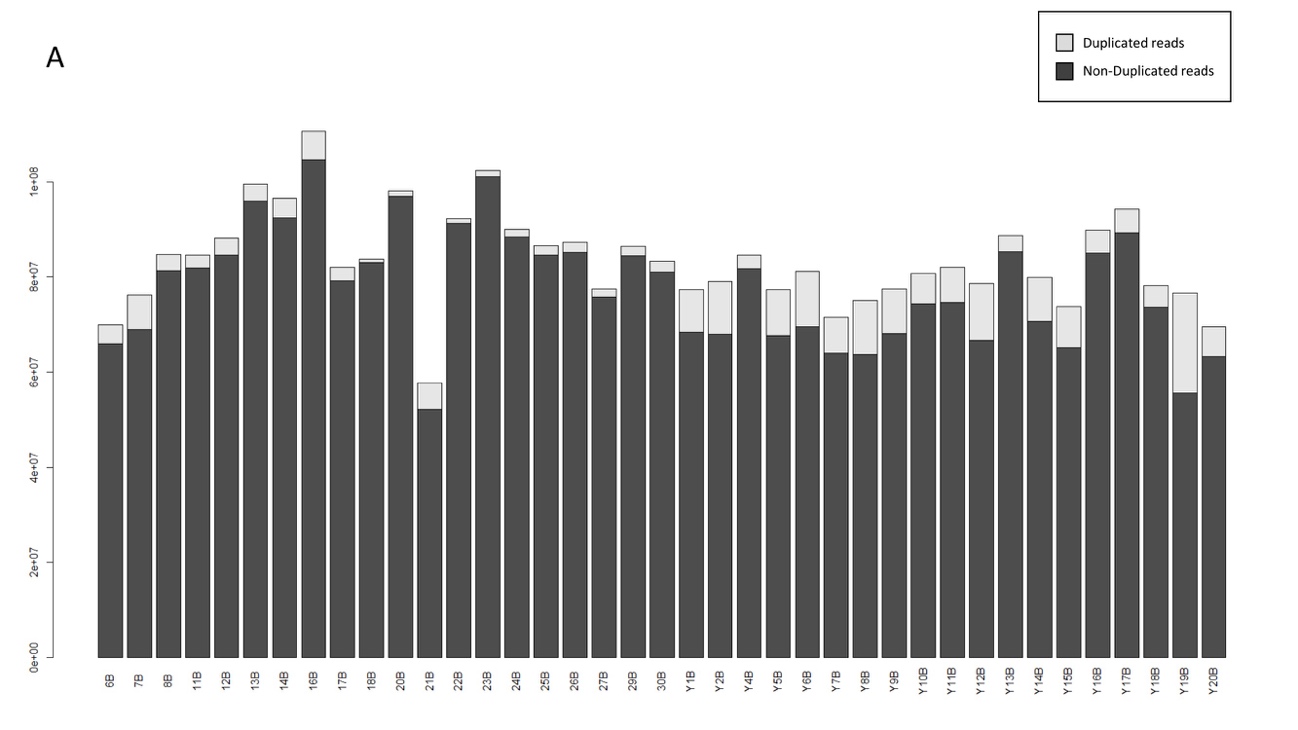

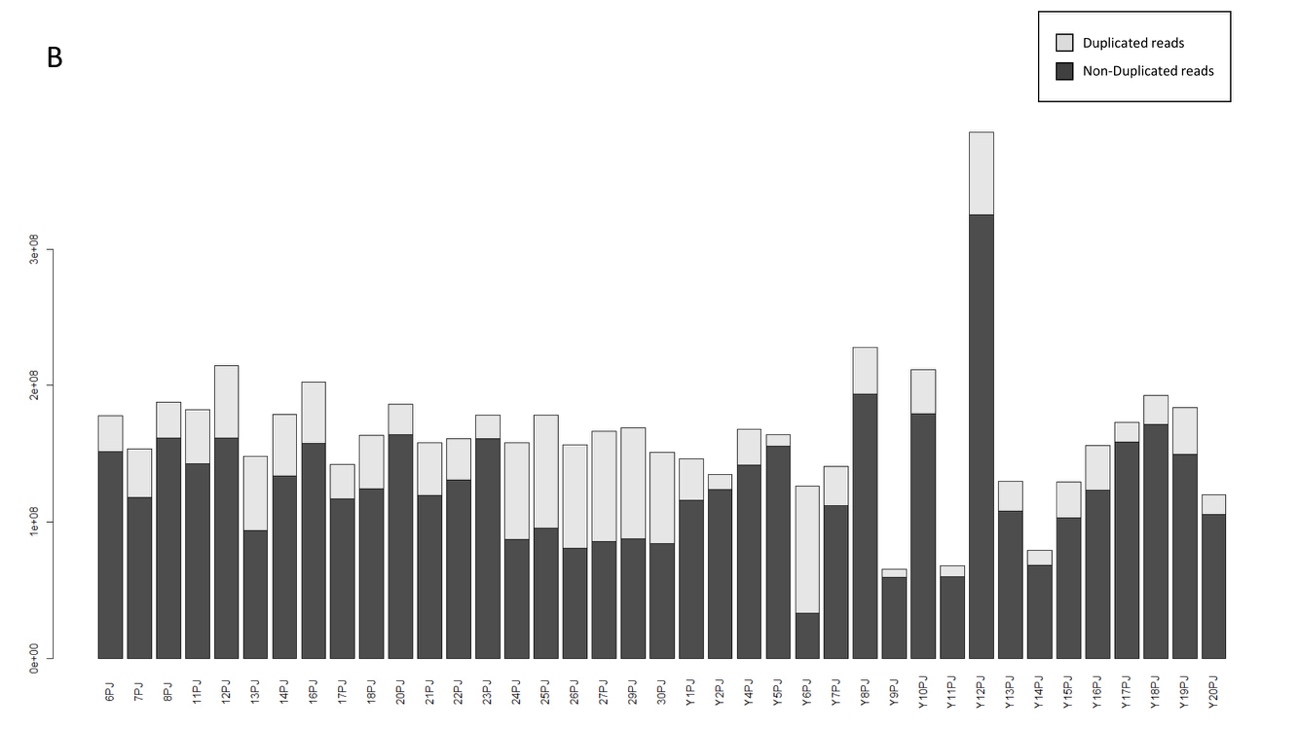
**

**Figure S5: Mutation patterns by histological Grade:** By merging the mutation pattern of each sample by grade, we were able to visualize the general distribution. Despite the increasing number of mutations, the general pattern remains similar during the development of malignancy.


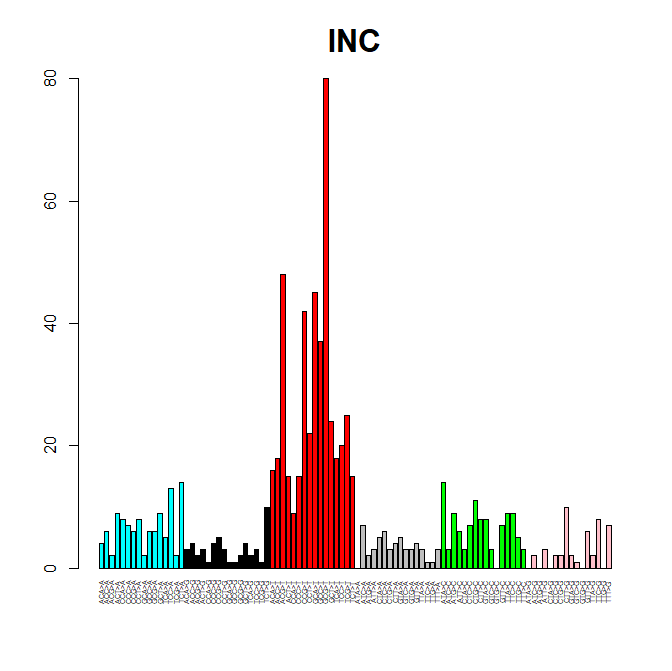

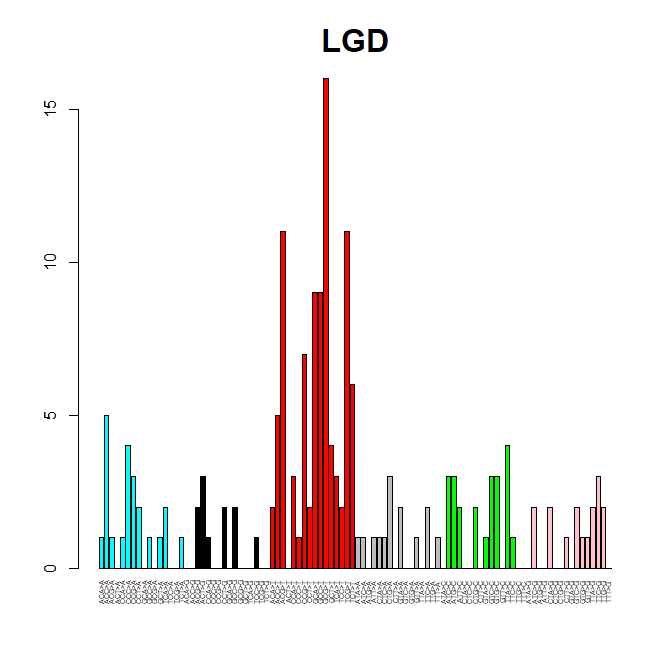

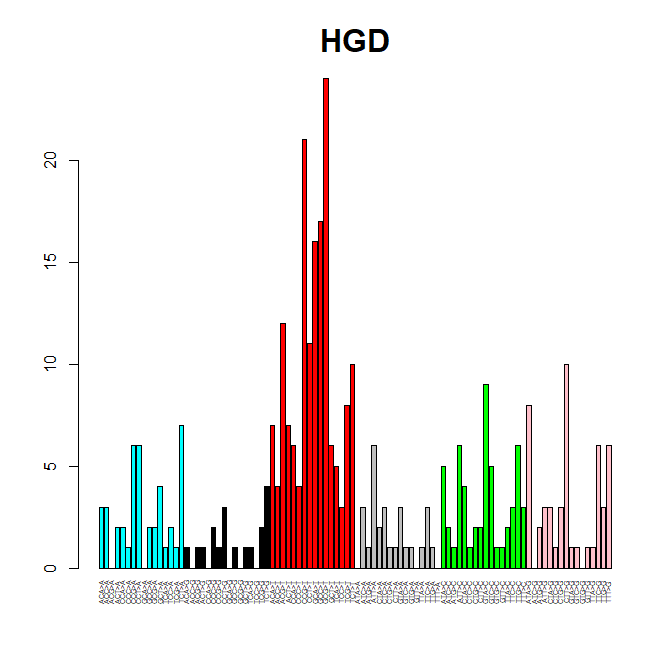


**Figure S6: Association study**. A: Heatmap containing the log of the p-values obtained Fisher test p-values showing the association between clinical data and SNV. B Association study between clinical data and CNA. All the p-values have been adjusted by adding one unit before the log transformation.


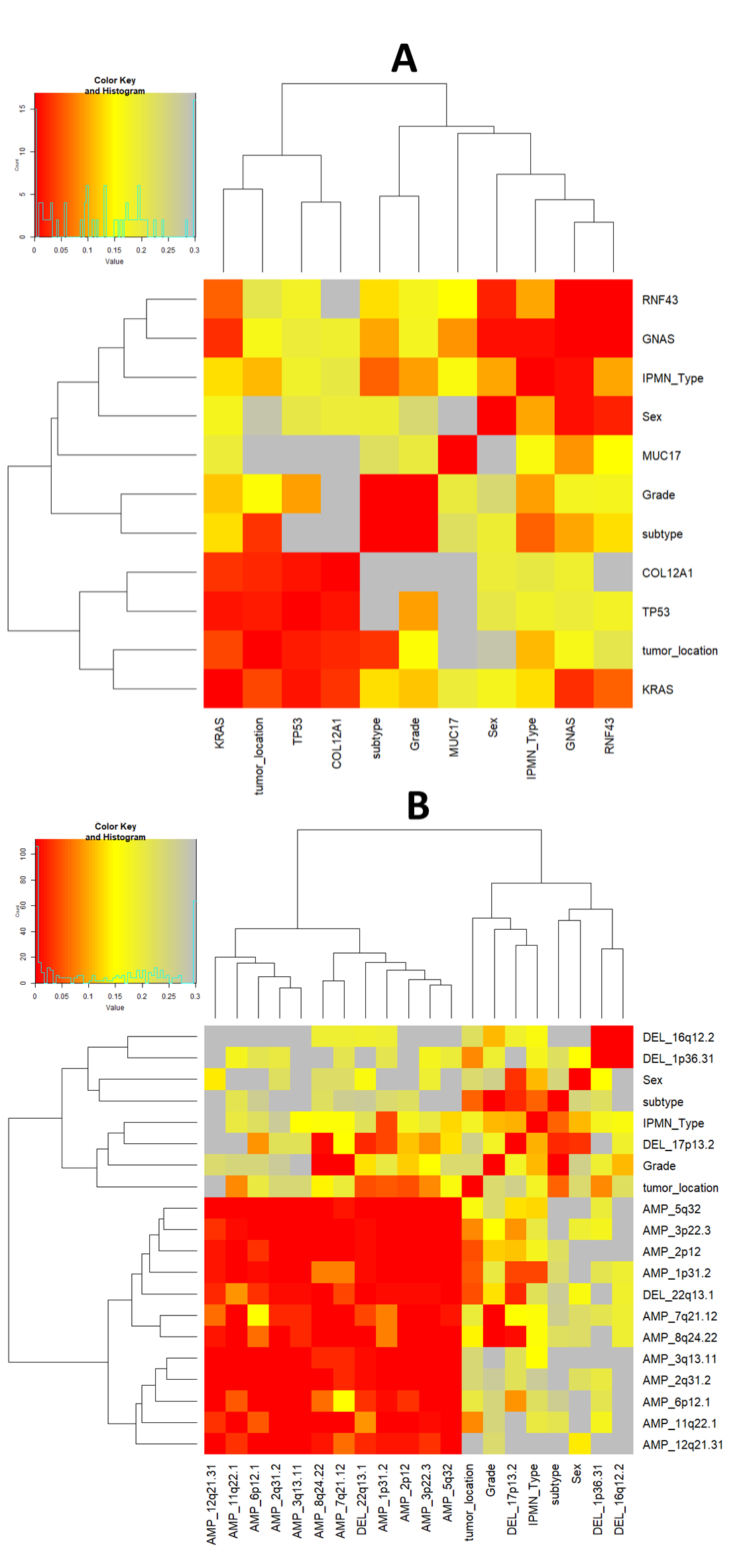


**Figure S7: SNV Validation.** The validation of the TP53 SNVs was done by capillary sequencing of DNA extracted from the FFPE specimens of the samples W14 (A) and W25 (B). TP53 mutations were both found in the malignant lesions (IC) and therefore validated.

**
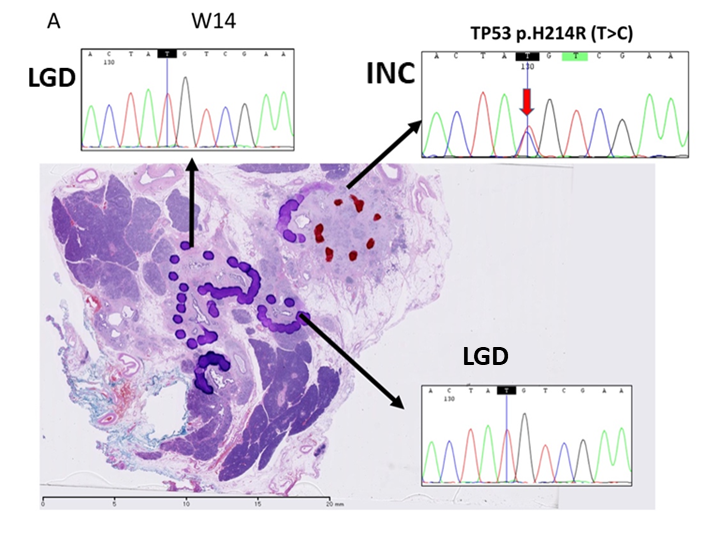
**


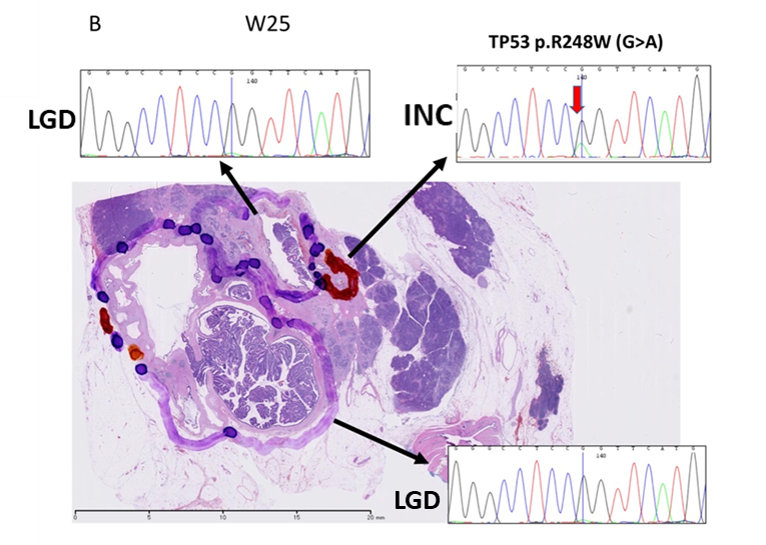


**Figure S8: Significantly deleted and amplified regions by grade.** By dividing our dataset in malignant (A), and benign (B), and applying GISTIC2.0 separately, we were able to observe that the deletion of 17p13.2 appears in the malignant set. This reinforces the association of TP53 with the development of malignancy in IPMN.


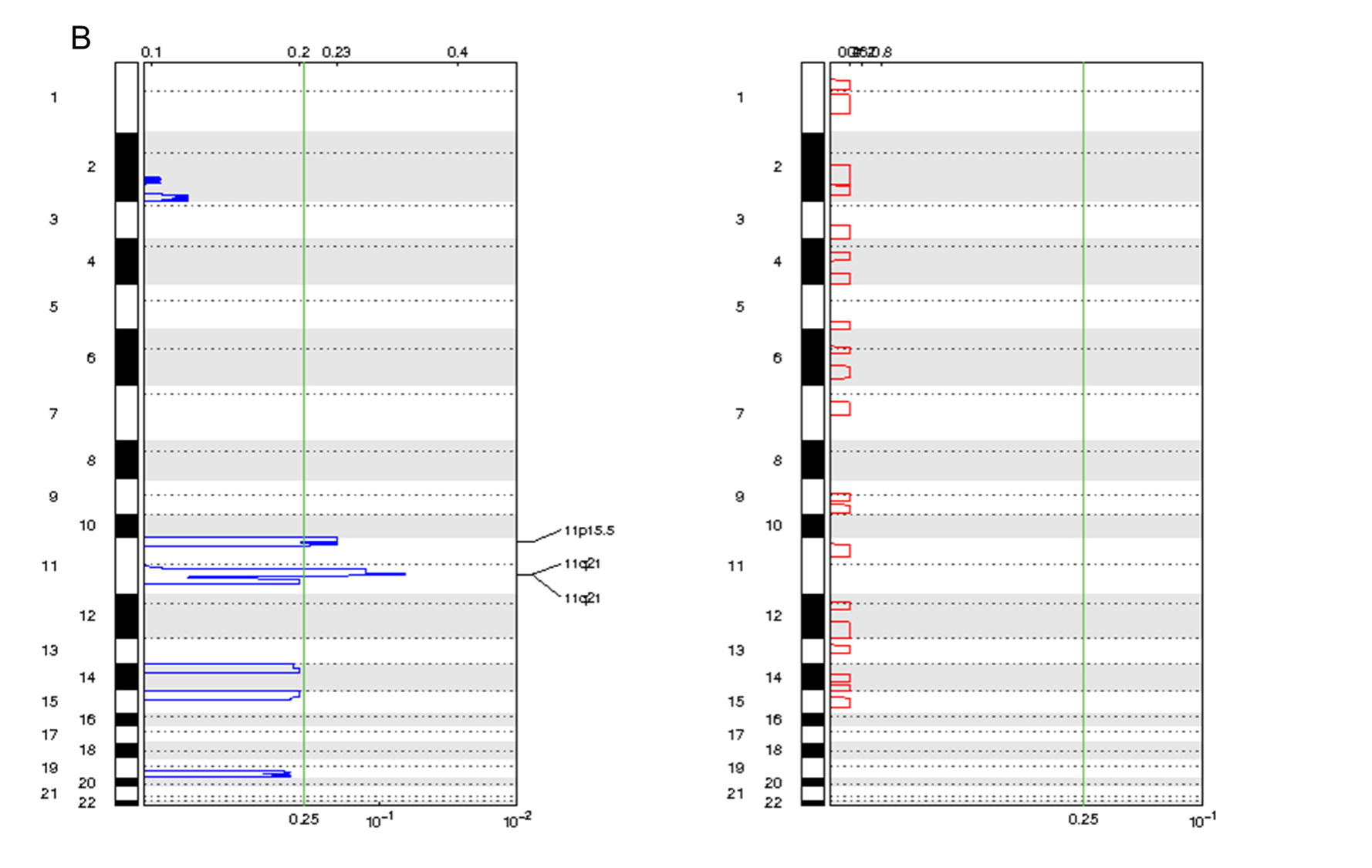

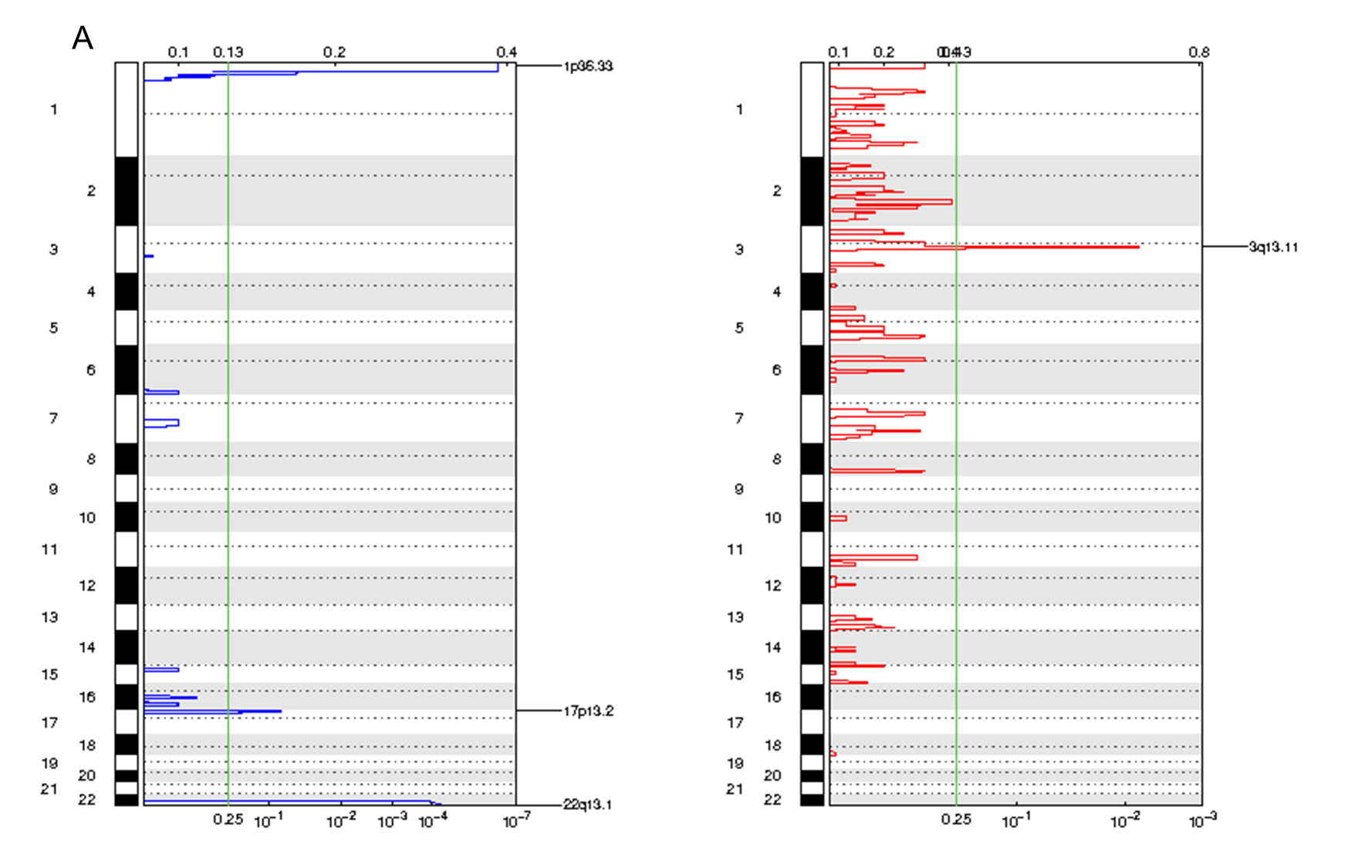

Supplement: Supplementary file 1 [file CAM4-8-4565-s001.docx]
